# Distributed cognition for collaboration between human drivers and self-driving cars

**DOI:** 10.3389/frai.2022.910801

**Published:** 2022-08-25

**Authors:** Alice Plebe, Gastone Pietro Rosati Papini, Antonello Cherubini, Mauro Da Lio

**Affiliations:** Department of Industrial Engineering, University of Trento, Trento, Italy

**Keywords:** autonomous driving, distributed cognition, human-vehicle collaboration, human-robot interaction, emergent behavior, artificial intelligence

## Abstract

This paper focuses on the collaboration between human drivers and intelligent vehicles. We propose a collaboration mechanism grounded on the concept of distributed cognition. With distributed cognition, intelligence does not lie just in the single entity but also in the interaction with the other cognitive components in a system. We apply this idea to vehicle intelligence, proposing a system distributed into two cognitive entities—the human and the autonomous agent—that together contribute to drive the vehicle. This account of vehicle intelligence differs from the mainstream research effort on highly autonomous cars. The proposed mechanism follows one of the paradigm derived from distributed cognition, the *rider-horse* metaphor: just like the rider communicates their intention to the horse through the reins, the human influences the agent using the pedals and the steering wheel. We use a driving simulator to demonstrate the collaboration in action, showing how the human can communicate and interact with the agent in various ways with safe outcomes.

## 1. Introduction

Recent developments in autonomous driving are leading to a transitional period, where human drivers and intelligent vehicles coexist. Nowadays, more and more commercial vehicles feature intermediate levels of automation. The presence of partially autonomous vehicles on the streets is starting to affect the traditional driver-vehicle interaction patterns. In fact, the addition of automation leads to a significant behavioral change in the way humans drive; interacting with partially automated systems disrupts the classic traffic dynamics, and it can cause unsafe interactions difficult to predict (Flemisch et al., 2017). Hence, the research community must place at the top of its agenda the issue of cognitive interaction between the driver and the automated system.

To date, research on vehicle intelligence has mainly addressed fully autonomous cars. They are far from the idea of human-vehicle collaboration, because the greater the automation, the less the human is involved in the driving task. In fact, the ideal self-driving vehicle would dispense with the human and any form of collaboration with them. The account of vehicle intelligence completely separated from the human driver has developed considerably, also because of the ongoing evolution of deep learning. However, the research is still far from achieving totally driverless vehicles, and it often overlooks the importance of mutual dependence between the human driver and the vehicle.

We argue that new forms of collaboration between humans and artificial agents can arise from the theoretical framework of distributed cognition, i.e., the idea to achieve a task through the emergent interaction of more intelligent entities. In the effort to achieve artificial driving agents with increasingly cognitive abilities, we see a promising direction in the idea of a distributed cognitive system: two cognitive entities—the human and the agent—collaborate to achieve the task of driving the vehicle. As we will show, this framework promotes new interesting ways to approach human-agent collaboration, leading to the formulation of a number of “metaphores” suggesting ideal styles of interaction.

We present a collaboration paradigm showing the advantages of having a system with more than a single cognitive entity. The system follows the *rider-horse* metaphor to implement distributed cognition. As the horse can “read” human's intentions and, reciprocally, the rider can understand animal's intentions, we argue that autonomous vehicles might benefit from a similar ability: the user experience would improve if the driver could give hints to the car and feel as if the car could “understand” their intentions. While the rider-horse system communicates with the reins, the human communicates with the agent using the pedals and the steering wheel. We show the collaboration system in action on a driving simulator. The results illustrate how the human can influence the agent's decision-making to obtain, for example, a lane change or an overtake whenever possible and safe; on the other hand, the agent can dismiss the human's suggestion if they are dangerous or not significant.

The following Section briefly introduces the different accounts of cognition proposed through the years, focusing especially on the distributed nature of cognition. Section 3 presents the main research direction pursued in autonomous driving, which sets aside the idea of collaboration with the human and focuses on vehicle intelligence as single cognition. Section 4 dives into the distributed account of vehicle intelligence and analyzes various collaboration paradigms between humans and autonomous agents. Section 5 presents the interaction mechanism we propose between a human and an autonomous agent previously developed. The section describes how the agent works (in brief) and how the interaction mechanism is realized. Section 6 demonstrates the system in action using a driving simulator. Lastly, Section 7 draws the conclusion and discusses future work.

## 2. Accounts of cognition

It already exists a form of intelligence capable of driving vehicles—humans. Thus, it is reasonable to design other forms of “vehicle intelligence” by taking inspiration from human intelligence and cognition. Human cognition is the focus of a vast area of research with a long-stand history. It is useful here to briefly sketch the different accounts of cognition proposed through the years, with special attention to the distributed nature of cognition.

One of the core ideas of cognitive science, at the time of its birth in 1956, is that minds and computers are exemplars of the same class, the *physical symbol system* (Gardner, 1985). A fundamental corollary of this theory is that what is possible for a human mind—for example, driving—is possible for a computer as well. This idea works in principle, but there is still no clear understanding on what kind of computations the human brain runs when, again for example, the person is driving a car.

Cognition has been characterized with a distributed structure since the early period of physical symbol. The “distributed” account of cognition mentioned so far considers only a single cognitive entity, composed of several sub-parts. However, there is cognition beyond the individual intelligence.

The idea of *distributed cognition* became popular with the work of Hutchins (1995b,a). This current of though stresses how the highest cognitive functions imply a strong social relation and cannot be studied in isolation (Cole and Engeström, 1993). Hutchins founded the concept of distributed cognition on his extended cognitive ethnography of ship navigation (Hutchins, 1995a): a ship requires a complex system made by both a team of people and an array of technologies, all working together. The team is organized, with precise roles for each crew member, and the cognitive work is offloaded thanks to aids such as instruments and charts. Hutchins further extended his study of distributed cognition from ship navigation to aviation—a domain closer to the focus of this paper than sea navigation (Hutchins, 1995b). The case analyzed by Hutchins is the management of the airplane's speed during landing. Speed is the most crucial factor for a safe landing. The process involves coordination within the crew as well as interaction with the instrumentation. Hutchins' account of cognition has been accepted as the best way to describe the dynamics and complexity of various human organizations, including classrooms, office work, company organization, and air traveling (Dror and Harnad, 2008).

Despite the innovation of Hutchins' work, cognitive science of that time was dominated by another school of though, called *4E Cognition* (Newen et al., 2018). The “4E” approach characterizes cognition with four features: *embodied, embedded, enacted*, and *extended*. Embodied cognition analyzes not just the role of the mind, but also the role the body has in cognition. Embedded cognition focuses on the integration of the cognitive agent into an environment. Enacted cognition assumes that knowledge is closely related to the notion of action (for example, perception is not just something propaedeutic to an action, it is a sort of action itself). We will not go in detail of these accounts of cognition as they are not the focus of the discussion; it is the last “E,” in fact, the most relevant in our context.

Extended cognition (Clark and Chalmers, 1998; Clark, 2008) presents an even more radical account of cognition than the one proposed by Hutchins. Extended cognition accepts as active components of cognition all kinds of things that can help humans think. Clark's famous example is the notebook used by a person suffering from memory loss. The person uses the notebook to take note of everything they need to know. For the person's cognition, the notebook plays a role as crucial and constitutive as their biological memory. Unlike Hutchins's, Clark's proposal spurred a huge debate within cognitive science (Menary, 2010), and it has become a key theoretical framework for topics such as the *Internet enhancement of cognition* (Smart, 2017). However, in the context of vehicle intelligence, Clark's notion of extended cognition is not so apt. According to him, the cognitive system gives equal partnership to the human mind and the external component. This aspect is questionable when the external part is trivially poor from the cognitive point of view—like the notebook example—or when the external part is a knowledge-packed resource like Wikipedia or Google.

**Figure 1:**
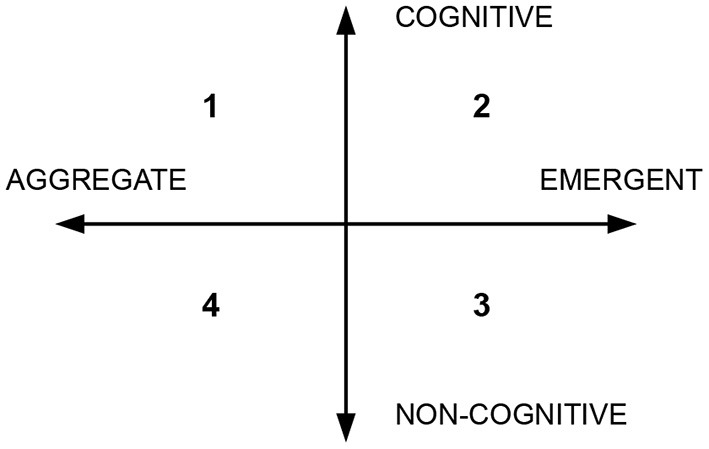
Conceptual space proposed by Poirier and Chicoisne (2008) to classify the relation between two entities in a cognitive system.

The cooperative relation between humans and their extended cognitive counterpart is well-represented in the framework proposed by Poirier and Chicoisne (2008). They present a two-dimensional conceptual space (see Figure 1) to classify the cooperation between two entities in a cognitive system. One axis represents the degree of cognition of the entities, ranging from *cognitive* to *non-cognitive*. For example, a pencil is totally non-cognitive, while humans have maximum degree of cognition. The other axis represents the outcome of the cooperation, which ranges from *aggregate* to *emergent*. Aggregate means there are no cooperative or inhibitory interactions among the parts of the system, and the task can be achieved even when parts of the system are removed. Emergent represents the opposite, when the parts of the system collaborate actively to achieve a shared task. For example, two researchers write a scientific a paper, and they agree to split the work in half; each person writes only a specific section of the paper without reading the rest. Only at the end, they merge the sections together. In this way, there is no cognitive advantage from the cooperation, because it is a simple aggregation of individual cognitive loads. Although the entities have high degree of cognition, the collaboration is aggregate. Any approach exploiting the concept of distributed cognition should fall in quadrant 2 of this conceptual space.

In the following sections, we will analyze current approaches to vehicle intelligence and how they relate to distributed cognition.

## 3. Vehicle intelligence with single cognition

**Figure 2:**
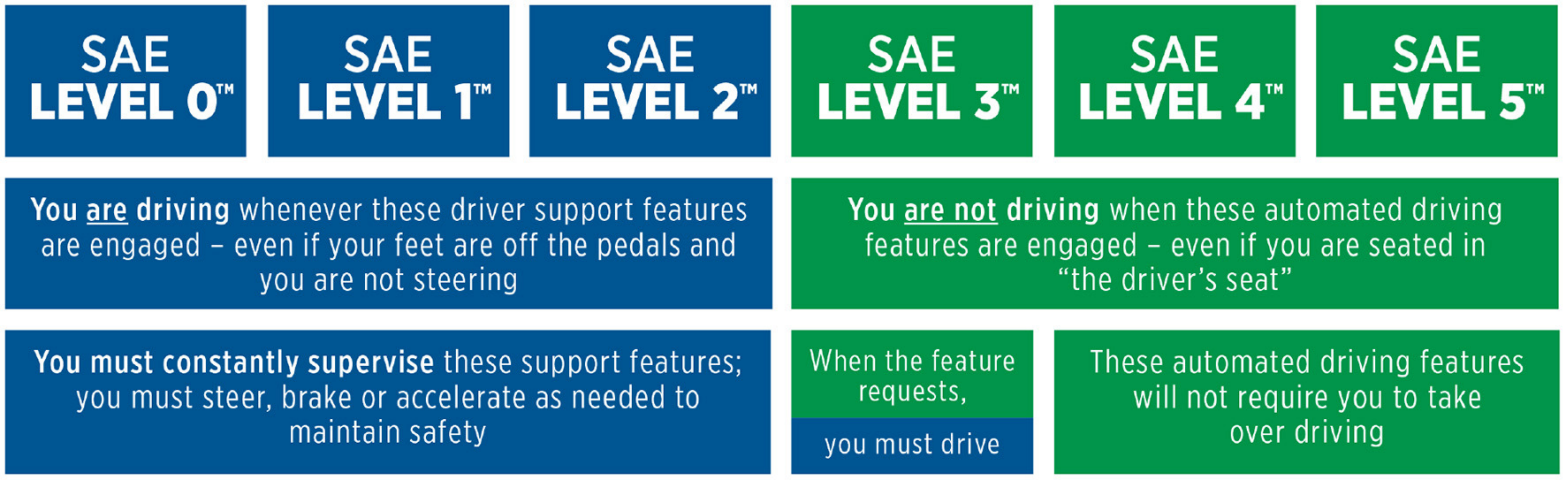
SAE levels of automation SAE (2021).

The main research direction in autonomous driving focuses on developing high levels of driving automation—the higher the level, the less the human is involved in the driving task. The Society of Automotive Engineers (SAE) defines six levels of driving automation (SAE, 2021), summarized in Figure 2. Level 0 stands for no automation at all, i.e., traditional automobiles. Level 1 introduces basic forms of driver assistance, such as emergency braking. Level 2, also called *partial automation*, is the form of automation currently available on recent vehicles, and it includes systems like adaptive cruise control and lane following. However, the human is still responsible of driving the car and must constantly supervise the system. Level 3, also called *conditional automation*, introduces a drastic shift from the previous level. Here, the system is responsible for driving the car and supervising the scene, while the human is allowed to engage in other activities. These systems operate in limited operational design domains, usually highways. Still, emergency situations might occur where the system is not able to proceed safely: in these cases, the system disengages from the driving task and requires the human to resume control of the vehicle with short time. Level 4 does not need human supervision. The system can work even without a person inside the vehicle. However, it still operates in limited domains. The operational domains usually consist of highway scenarios, which are easier to manage with respect to urban scenarios. Driving in urban areas presents a bigger challenge because of multiple traffic directions, intersections, parked vehicles, traffic lights, sidewalks, and numerous classes of *vulnerable road users*. Lastly, Level 5 represents full automation—ideally, a car without steering wheel and pedals. Here, the human driver is completely replaced by the system, which is able to operate in any conditions without limitations.

Most of the research effort is now put into developing Levels 4 and 5. This research direction overlooks the idea of collaboration between human and driving agent. The vehicle intelligence, in this account, aims to gradually assume the role of the driver and make the human simply a passenger not involved in the driving task. In fact, the higher the level of autonomy, the more the human driver is replaced by the artificial agent. This approach to autonomous vehicles is far from distributed cognition—there are two cognitive entities in the system, but there is no collaboration between them. Either the agent or the human is in charge of controlling the vehicle, and when necessary the control passes to one another. Disengagements, i.e., where the human must resume control of the vehicle because the agent stops working safely, are one of the most critical aspects of autonomous driving systems. The problem of disengagements affects Levels 3 and 4 the most, precisely because the collaboration between the cognitive entities in the system is missing. In fact, the more automation is added to the system (and the more reliable and robust the autonomy is), the human is less likely to predict the automation failure dues to the lack of cognitive engagement (Endsley, 2017). For this reason, Levels 3 and 4 are paradoxically less reliable than Level 2, where the human should be constantly supervising the system (however human beings tend to misuse Level 2 not supervising as they should).

Level 5 of autonomy can be the solution to the conundrum of disengagements, since the system would never require the person to take over driving. Completely replacing human drivers with artificial drivers is indeed desirable, but still a challenging task. Production-level deployment of full self-driving vehicles remains a distant future (Jain et al., 2021). On the one hand, state-of-the-art driving agents surpass humans in computation, responsiveness, and multitasking. On the other hand, humans exceed automation in the capacity of detection, context understanding, induction, and improvisation (Xing et al., 2021). For this reason, researchers are looking at new directions to develop Level 5 systems focusing on cognitive-inspired approaches. To achieve an AI capable of handling any possible (or unseen) traffic scenario, it appears more and more necessary to develop high-level cognitive abilities similar to humans (Wang et al., 2021). Implementing human-like cognitive behaviors is far from easy. As discussed in Section 2, there are countless theories trying to progress the understanding of the mind and the brain. The current understanding of how the brain executes complex behaviors such as driving is vague, often controversial, and short of detail.

Given the challenges linked to Level 5 systems, a parallel research direction looks at the concept of distributed cognition applied to vehicle intelligence. The idea is to design systems where the collaboration between human and agent is at the core of the driving mechanism. This approach takes the best of both worlds, leveraging the potential of human intelligence and the computational power of machine intelligence.

## 4. Vehicle intelligence with distributed cognition

As mentioned in the Section 1, vehicle automation will cause significant behavioral changes in human driving. The behavioral change depends on the way vehicle intelligence is designed. In the “single cognition” account reviewed in Section 3, humans are gradually removed from the driving task. However, the transition from Levels 2–3 to Levels 4–5 is proceeding slowly, forcing human drivers to interact with partially automated systems—often without being aware that other vehicles are controlled by artificial agents. These interactions disrupt the classic traffic dynamics and can produce unsafe scenarios (e.g., disengagements) that are difficult to predict (Flemisch et al., 2017).

The “distributed cognition” account of vehicle intelligence approaches the problem of driver-vehicle interaction patterns differently. Cooperative vehicle intelligence is grounded on the idea that, in a system, knowledge does not lie solely within the individual but rather within all entities involved in the system (Banks and Stanton, 2017). This follows Hutchins' account of distributed cognition, described in Section 2. Applying this idea to intelligent vehicles means that humans and driving agents must collaborate actively. The driving task is achieved only through the interaction of the two entities, because each contributes with a different (if not complementary) set of cognitive skills.

It is not straightforward to determine how the skills of drivers and automated vehicles can be combined for optimal cooperation. Researchers have proposed metaphors to extract design concepts for ideal human-agent interaction. Marcano et al. (2020) pinpoint four metaphors used as “blueprint” for distributed driving systems. The first is the *rider-horse* metaphor, also called *H-Metaphor* (Flemisch et al., 2003). It compares the human-agent interaction to a human riding a horse. When riding, the human controls the horse through the reins. This haptic interface allows the horse and the rider to “understand” each other's intentions. In addition, the rider can take the horse under tight reins to exert more direct control or can use loose reins to provide the horse with a higher degree of autonomy. The second metaphor is the *aviator instructor-student* (Holzmann et al., 2006). It describes the interaction occurring in a flying training session between a student and an experienced aviation pilot. The expert aviator assists the beginner either actively (by exerting forces on the control system) to help with the execution of maneuvers, or passively (by holding the steering control with different forces) to approve or disapprove the student's action. The next metaphor is the *joint-carrying* of an object (Flemisch et al., 2016). It emphasizes the collaboration between two agents that share the same task and interact physically on the same object. The interesting aspect is that the agents have different perception capabilities—in the specific example, one is walking forward and the other backwards. Yet, the information perceived by an agent complements each other, and both are needed to complete the task. Lastly, the *parent-child* metaphor illustrates a parent teaching a child to ride a bike (Flemisch et al., 2012). In this metaphor, the child has control of the bike, and the parent does not interfere while the child is performing well. If the child starts wobbling, the parent intervenes in proportion to the risk—the intervention should be gentle in any case, to avoid rejection of the assistance.

All metaphors are relevant to the case proposed here, but with various degrees. The least relevant metaphor is the *parent-child*, while it is certainly true that the autonomous system should avoid to overwhelm drivers while they are performing well, and gently intervene if the driver leads the vehicle to an unsafe condition. The *joint-carrying* metaphor describes well one specific aspect: the different and complementary perceptions of the scene by the driver and the system. However, it goes no further in indicating how these differences should be reconciled. The *aviator instructor-student* metaphor brings us back into the domain of aviation, which has certain affinities with autonomous driving, as commented in Section 2. Aeronautics has a long history of automated procedures and human-computer interactions. However, there are obvious differences with respect to autonomous driving. For example, in the context of airplanes, distributed cognition implies a distribution of roles within the crew, while this is irrelevant in an autonomous car. Moreover, a vehicle continuously interacts with the environment and the other road users at close range. On the other hand, there are lessons that can be taken from the field of aviation. As the role of the driver becomes gradually closer to that of an airplane pilot, a new class of errors can lead to incidents. In aviation, a *classification error* occurs when the pilot assumes that the system is working in a way that is different from the actual state of the system. This form of error seems likely to occur within driving automation as well—this is discussed in more detail in Banks and Stanton (2017, p. 15–16). It is, however, the *rider-horse* metaphor that captures in the best and most complete way the current proposal, as we will explain in Section 5.

Reviews on driver-vehicle collaboration can be found in Xing et al. (2021), Marcano et al. (2020), Bengler et al. (2014), and Michalke and Kastner (2011). Works focus on key factors like human trust and situation awareness, which influence the design of the system. Moreover, the form of interaction defines the control mechanism—we can distinguish between shared control and take-over control. The type of control mechanism determines also how to implement the steering/pedal system, either with a coupled or uncoupled control framework. However, not all attempts at driver-vehicle collaboration can be considered forms of distributed cognition. Recalling the diagram of Figure 1 proposed by Poirier and Chicoisne (2008), there are approaches that fall outside quadrant 2, which is the only quadrant identifying distributed cognition. Consider, for example, low-level ADAS systems such as emergency brake or lane departure warning: they have very low degree of cognition. Hence, it is not possible to talk about distributed cognition—they belong to quadrant 4. On the other hand, more advance (cognitive) systems like overtaking assistance tends to generate aggregate outcomes, as opposite of emergent outcomes according to the classification of Poirier and Chicoisne (2008). In these systems, there is no overt cooperative interactions between the human and the assistant: the assistant is either on or off, and there is no mean of communication between the parts. Moreover, when the assistant is off, the human can still achieve the driving task. Therefore, the systems are not emergent and fall in quadrant 1.

In the next section, we will describe our implementation of distributed cognition in an autonomous driving system.

## 5. Methodology

We present a collaboration paradigm between a self-driving agent and a human driver based on the H-metaphor. In the proposed system, just like the rider influences the horse's behavior using the reins, the human driver steers the decision-making of the autonomous agent using the pedals and the steering wheel. The system uses an uncoupled control framework and is an example of distributed cognition applied to vehicle intelligence. The two entities in the system are both intelligent, both interpreting the world, and working jointly to achieve the same task. The self-driving agent has high degree of cognition and collaborates with the human in an emergent way. Therefore, the proposed systems falls in quadrant 2 of the classification space of Poirier and Chicoisne, in Figure 1.

The autonomous agent considered here has been developed within the European H2020 project Dreams4Cars^1^. The agent has the cognitive capabilities necessary to drive a vehicle autonomously, in controlled situations, and it can be regarded as Level 4 in the SAE definition. Note that this work focuses on the interaction paradigm between the agent and the human, rather than how the autonomous agent works. Here, we include only a brief explanation of the agent to better understand the collaboration mechanism; a detailed description of the agent architecture is in Da Lio et al. (2020).

The sensorimotor system of the agent is designed to be compatible with the human system. Specifically, the agent must be capable of seeing the action possibilities latent in the environment—dubbed *affordances* by Gibson (1986)—and it has to generate the corresponding action plans in a way similar to a human driver. An example of affordance, taken from Gibson's original work, is the vision of a stair; it elicits the action of stepping, up or down, relative to the size of the person's legs. Another example—very close to the problem under consideration here—is the following: “*The progress of locomotion is guided by the perception of barriers and obstacles, that is, by the act of steering into the openings and away from the surfaces that afford injury”* (Gibson, 1986, p. 132). For a self-driving agent, the affordances are the physically traversable space constrained by traffic rules and space-time restrictions from moving obstacles. The rider-horse collaboration has the same scheme, since the horse sees the same affordable paths of the rider, and the rider can infer the horse's intentions.

The agent works in two phases: action priming and action selection. During action priming, the agent detects the set of affordances *D* in the navigable space and maps them onto estimates of their *salience*. The salience measures how good the corresponding action is. The actions that the agent can produce are the set of trajectories *U* (i.e., time-space locations of the vehicle) that originate from the current configuration. Since a vehicle has two controllable degrees of freedom, the whole space of possible actions is spanned by the specification of the longitudinal and lateral controls. In our implementation, the longitudinal control is the jerk *j*, and the lateral control is the steering rate *r* (i.e., the time derivative of the steering angle). For an instantaneous action *u* = 〈*j, r*〉, ν(*u, d*) represents how good or desirable the action is in relation to the affordance *d*. ν(*u, d*) evaluates two factors: the probability of remaining in the specified spatial domain of the affordance *d* for a sufficient time; the travel time subject to speed limits and comfort criteria. The salience to express how good the choice of the current control 〈*j, r*〉 for the affordance *d* is the following:


(1) $$\begin{array}{r} s_{d}\left(j,r\right)=\underset{u\in U}{\sup}\left\{\nu \left(u,d\right)\right\}. \end{array}$$

This means that the salience of the instantaneous choice 〈*j, r*〉 for the affordance *d* is the value ν(ũ, *d*) of the optimal action ũ among all actions beginning with 〈*j, r*〉. The global salience function can be defined as follows, where weights *w*_*d*_ serve to prioritize sets of affordances:


(2) $$\begin{array}{r} s\left(j,r\right)=\underset{d\in D}{\max}\left\{w_{d}s_{d}\left(j,r\right)\right\}. \end{array}$$

During the second phase of action selection, the agent chooses the motor control 〈*j, r*〉 corresponding to the maximum salience and executes it.

The way the autonomous agent works is broadly inspired by how human cognition (presumably) realizes the driving task. Hence, it is reasonable to expect that a similar process occurs in the mind of the human inside the vehicle the agent is controlling. The human recognizes their own set of affordances and computes a salience value s^*^(*j, r*) for each action they have in mind. We assume that the sensorial system of the vehicle is reliable—as it is indeed in most situations—and that the system has learned an efficient control policy in response to affordances. Hence, is it reasonable to expect in most cases that s^*^(*j, r*)≈*s*(*j, r*). For a more detailed explanation, see Da Lio et al. (2017). However, there can be situations where the person desires a different action or have a specific goal in mind the agent is not aware of. With distributed cognition, the human can obtain the desired behavior by collaborating with the agent, which is able to interpret the human's intention.

The human interacts with the agent by biasing the action selection process, through the pedals and the steering wheel. The gas/brake pedals control the longitudinal bias, and the steering wheel controls the lateral bias. The biases influence the computation of the global salience (2) by applying weights either to sets of affordances or to individual ones. In the case of longitudinal bias, the human can suggest the agent to drive faster/slower by pressing the gas/brake pedal—hence, applying a weight to the faster/slower affordances. The modified salience function is the following:


(3) $$\begin{array}{r} s^{\prime }\left(j,r\right)=k\left(g-b\right)js\left(j,r\right) \end{array}$$

where *g* and *b* are the normalized gas and brake strokes, and *k* is a convenient gain. In the case of lateral bias, the human can prompt the agent to change lane to the left/right by steering the wheel. This action weights the individual affordances corresponding to lane change in the suggested direction.

The presented collaboration paradigm works safely because of distributed cognition. Since the system is composed of two cognitive capable entities, each of them can supervise the other and prevent wrong behaviors. For example, if the human suggests to perform a dangerous or unfeasible maneuver, the agent ignores the command. In fact, the agent dismisses any action that is not affordable or for which the salience is low or inhibited. This mechanism is one of the most critical part in the system; Section 7.1 further analyzes its limitations and how to resolve them. The next section provides simulations demonstrating these safe behaviors.

## 6. Demonstrations

**Figure 3:**
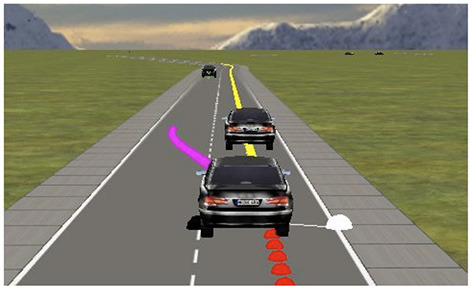
Screenshot of the OpenDS simulator showing the human biasing the agent to overtake. The scenario corresponds to Figure 4(i), showing the affordances a in yellow and b in purple.

We test the collaboration mechanism in the open-source driving simulator called OpenDS^2^, depicted in Figure 3. This Section describes the outcome of five tests carried out in three simulated scenarios. In each test, we focus on how the human can promote various driving actions by collaborating with the autonomous agent.

**Figure 4:**
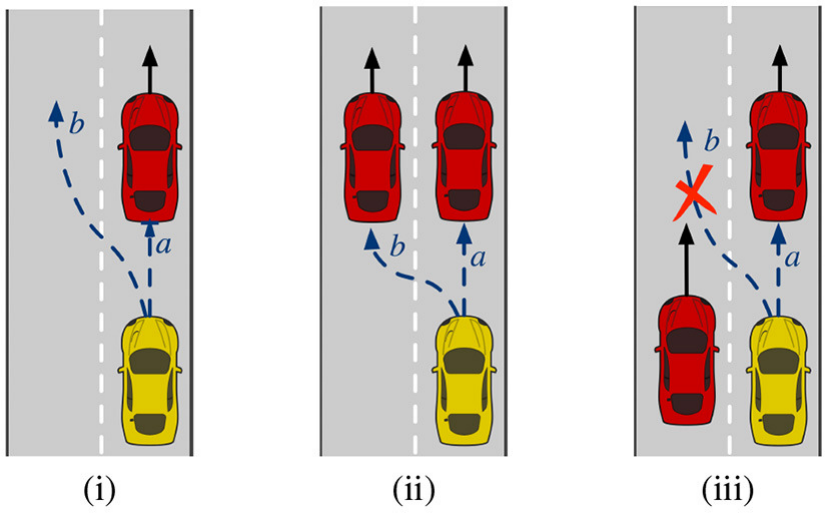
Three simulated scenarios to test the collaboration between the agent and the human (yellow car) when other vehicles are present (red cars). The dashed arrows show the affordable actions. **(i)** It is possible to follow the red car or to overtake. **(ii)** It is possible to follow the car on the right or the one on the left. **(iii)** The only affordable action is to follow the car ahead.

The first test scenario shown in Figure 4(i) is a two-lane straight road where overtaking is possible and safe. In the same lane, there are the ego-vehicle and another car ahead of it, colored in yellow and red respectively. The autonomous agent identifies two possible affordances: to follow the red car (affordance a in the figure), or to overtake (affordance b). Since the leading vehicle is driving at almost the speed limit, the agent chooses the affordance a. When the human steers the wheel to the left, they exert a bias toward the affordances corresponding to the left lane (just b in this example). As a result, the salience of affordance b surpasses a, and the agent executes the action of overtaking the red car. A second test with the same scenario demonstrates the same outcome but with a different form of interaction. This time, the human promotes the overtake by pressing the gas pedal rather than steering the wheel. The positive bias affects the weights of faster affordances, that is b. Hence, just like before, the agent shifts from a to b and overtakes the leading car.

In the next scenario, Figure 4(ii), there are two cars ahead of the ego-vehicle, which occupy both lanes and travel at the same speed. In this case, affordances a e b have the same longitudinal control, i.e., b is not faster than a as before. The salience of a is grater than b because the latter discounts the cost of changing lane. Hence, the agent chooses a. If the human steers the wheel to the left, choosing the affordance b does not lead to any tangible speed improvement. However, the agent understands the human's desire and moves to the adjacent lane and starts following the left car. Using the same scenario, another test shows what happens if the human tries to bias the agent using the gas pedal rather than the steering wheel. In this case, the human bias has no effect because there are no affordable faster actions. The cars ahead do not allow the agent to increase the speed. Therefore, the human request cannot be satisfied, and agent keeps affordance a.

The last scenario, Figure 4(iii), shows a car ahead of the ego-vehicle and a car overtaking on the left lane. Here, affordance b no longer exists: the car on the left prevents the agent from changing lane. Since there are no affordable actions linked to the left lane, when the human steers the wheel of presses the gas pedal, there is no effect. The agent ignores the human's request and remains on the right lane following a. Further simulations are available in Da Lio et al. (2022).

## 7. Discussion

In this paper, we have argued that vehicle intelligence can benefit from the theoretical concept of distributed cognition. Distributed cognition can help designing new paradigms of collaboration between human drivers and autonomous agents. Cooperative vehicle intelligence is grounded on the idea that, in a system, knowledge does not lie solely within the individual but rather within all entities involved in the system. This research line moves away from the mainstream development of autonomous driving, which aims to completely remove humans from the driving task. However, fully autonomous vehicles are still far from being achieved, while distributed vehicle intelligence can solve at the present time the problems caused by the disruption of classical traffic dynamics.

We have proposed a collaboration paradigm founded upon the rider-horse metaphor, allowing the human to influence the decision making of the driving agent. Just like the rider communicates their intention to the horse through the reins, the human interacts with the agent using the pedals and the steering wheel. If the human presses the gas/brake pedal, they suggest the agent to drive faster/slower. If the human steers the wheel, they suggest the agent to change lane.

The collaboration system can support the user also in situations where the human is normally uncertain on how to behave. For example, the user hesitates to overtake because they are not sure about the feasibility of the maneuver. The user may want to drives closer to the opposite lane to see better ahead before deciding whether to overtake. With our collaboration mechanism, the user is not responsible to evaluate if it is safe or not to overtake. It is the agent that performs the evaluation and, in positive case, executes the overtake. Therefore, there is no need anymore for the user to drive for a moment to the center to have a clearer view of the road, because the agent is the one responsible to check if the overtake is feasible. Even if the user steers to the side to see ahead, the agent will not execute the overtake if it deems the maneuver risky (note that the perception system of the agent differs from the human, so the agent does not actually need to drive to the side to see better, like a human driver would do—although there are new attempts at human-inspired perception for autonomous vehicles; Plebe et al., 2021). This collaboration paradigm leads to a new way of driving, and human drivers will need some time to adjust to it. With this collaborative style of driving, humans, and agents become responsible for decisions at different levels: the agents take care of the execution of safe maneuvers, and the humans decide on the overall driving style, e.g., faster/slower, conservative/aggressive, and such.

### 7.1. Limitations and future work

The distributed cognition approach works best when the entities in the system lie close in the “cognitive” axis of the classification space of Figure 1. In the context of vehicle intelligence, this means that the human and the driving agent should be capable of understanding each other's intentions. In other words, they should share the same affordances. Unfortunately, this is not always the case. Fully autonomous and reliable driving agents do not exist, yet. Hence, unpredictable situations are still possible, where the human detects unconventional affordances that the autonomous agent is not aware of. For example, if the road is blocked by a tree, an autonomous agent would stop forever; however, a human driver could be aware that the ground on the side of the road is “driveable” and that it is possible to bypass the blockage by driving on the gravel. This is an affordable action that the agent would most likely miss.

Future work is to extend the collaboration paradigm with a “tight reins” mode—to use an expression in accordance with the H-metaphor. With this mode, the human can apply a tight control to make the agent accepts affordances not known before and generate new behaviors. If the user insists on an action that the agent is refusing to perform because not affordable in its view, after a certain “persistence threshold”, the agent accepts the new affordance and executes the maneuver. A similar concept can be found in Vanholme et al. (2011) for driving on highways. This solution would mitigate the issue of artificial systems dangerously overriding human decisions—an issue common also to other research domain such as aeronautics.

## Data availability statement

The original contributions presented in the study are included in the article/supplementary material, further inquiries can be directed to the corresponding author/s.

## Author contributions

AP wrote the manuscript with input from all authors. GR and AC carried out the simulations. MD was in charge of overall direction and planning. All authors contributed to the article and approved the submitted version.

## Conflict of interest

The authors declare that the research was conducted in the absence of any commercial or financial relationships that could be construed as a potential conflict of interest.

## Publisher's note

All claims expressed in this article are solely those of the authors and do not necessarily represent those of their affiliated organizations, or those of the publisher, the editors and the reviewers. Any product that may be evaluated in this article, or claim that may be made by its manufacturer, is not guaranteed or endorsed by the publisher.

## Footnotes

^1^ https://www.dreams4cars.eu

^2^ https://opends.dfki.de/

## References

[B1] Banks V. A Stanton N. A. (2017). Automobile Automation: Distributed Cognition on the Road (1st ed.). Boca Raton, FL: CRC Press. 10.1201/9781315295657

[B2] Bengler K. Dietmayer B. Maurer M. Stiller C. Winner H. (2014). Three decades of driver assistence systems. IEEE Intell. Transp. Syst. Mag. 6, 6–22. 10.1109/MITS.2014.233627131174275

[B3] Clark A. (2008). Supersizing the Mind. Oxford: Oxford University Press. 10.1093/acprof:oso/9780195333213.001.0001

[B4] Clark A. Chalmers D. (1998). The extended mind. Analysis 58, 7–19. 10.1093/analys/58.1.7

[B5] Cole M. Engeström Y. (1993). “A cultural-historical approach to distributed cognition,” in Distributed Cognitions: Psychological and Educational Considerations, ed G. Salomon (Cambridge, UK: Cambridge University Press), 916–945.

[B6] Da Lio M. Doná R. Rosati Papini G. P. Gurney K. (2020). Agent architecture for adaptive behaviors in autonomous driving. IEEE Access 8, 154906–154923. 10.1109/ACCESS.2020.3007018

[B7] Da Lio M. Doná R. Rosati Papini G. P. Plebe A. (2022). The biasing of action selection produces emergent human-robot interactions in autonomous driving. IEEE Robot. Autom. Lett. 7, 1254–1261. 10.1109/LRA.2021.3136646

[B8] Da Lio M. Mazzalai A. Gurney K. Saroldi A. (2017). Biologically guided driver modeling: The stop behavior of human car drivers. IEEE Trans. Intell. Transp. Syst. 19, 2454–2469. 10.1109/TITS.2017.2751526

[B9] Dror I. E. Harnad S. (eds.). (2008). Cognition Distributed: How Cognitive Technology Extends Our Minds. Amsterdam: John Benjamins. 10.1075/bct.16

[B10] Endsley M. R. (2017). From here to autonomy: lessons learned from human-automation research. Hum. Factors 59, 5–27. 10.1177/0018720816681350 28146676

[B11] Flemisch F. Abbink D. Itoh M. Pacaux-Lemoine M.-P. Weßel G. (2016). Shared control is the sharp end of cooperation: towards a common framework of joint action, shared control and human machine cooperation. IFAC-PapersOnLine 49, 72–77. 10.1016/j.ifacol.2016.10.464

[B12] Flemisch F. Altendorf E. Canpolat Y. Weßel G. Baltzer M. Lopez D.. (2017). “Uncanny and unsafe valley of assistance and automation: First sketch and application to vehicle automation,” in Advances in Ergonomic Design of Systems, Products and Processes, ed T. Inagaki (Cham: Springer), 319–334. 10.1007/978-3-662-53305-5_23

[B13] Flemisch F. Heesen M. Hesse T. Kelsch J. Schieben A. Beller J. (2012). Towards a dynamic balance between humans and automation: authority, ability, responsibility and control in shared and cooperative control situations. Cogn. Technol. Work 14, 3–18. 10.1007/s10111-011-0191-6

[B14] Flemisch F. O. Adams C. A. Conway S. R. Goodrich K. H. Palmer M. T. Schutte P. C. (2003). The H-Metaphor as a Guideline for Vehicle Automation and Interaction. Technical report. NASA.

[B15] Fodor J. (1983). Modularity of Mind: and Essay on Faculty Psychology. Cambridge, MA: MIT Press. 10.7551/mitpress/4737.001.0001

[B16] Gardner H. (1985). The Mind's New Science-A History of the Cognitive Revolution. New York, NY: Basic Books.

[B17] Gibson J. (1986). “The theory of affordances,” in The Ecological Approach to Visual Perception (Mahwah, NJ: Lawrence Erlbaum Associates), 127–143.

[B18] Holzmann F. Flemisch F. O. Siegwart R. Bubb H. (2006). From Aviation Down to Vehicles-Integration of a Motions-Envelope as Safety Technology. Technical report, SAE Technical Paper. SAE International. 10.4271/2006-01-1958

[B19] Hutchins E. (1995a). Cognition in the Wild. Cambridge, MA: MIT Press. 10.7551/mitpress/1881.001.0001

[B20] Hutchins E. (1995b). How a cockpit remembers its speeds. Cogn. Sci. 19, 265–288. 10.1207/s15516709cog1903_1

[B21] Jain A. Del Pero L. Grimmett H. Ondruska P. (2021). Autonomy 2.0: why is self-driving always 5 years away? arXiv preprint arXiv:2107.08142. 10.48550/arXiv.2107.08142

[B22] Marcano M. DIaz S. Perez J. Irigoyen E. (2020). A review of shared control for automated vehicles: theory and applications. IEEE Trans. Hum. Mach. Syst. 50, 475–491. 10.1109/THMS.2020.3017748

[B23] Menary R. (ed.). (2010). The Extended Mind. Cambridge, MA: MIT Press. 10.7551/mitpress/9780262014038.001.0001

[B24] Michalke T. Kastner R. (2011). The attentive co-pilot: towards a proactive biologically-inspired advanced driver assistance system. IEEE Intell. Transp. Syst. Mag. 3, 6–23. 10.1109/MITS.2011.941911

[B25] Minsky M. (1986). The Society of Mind. New York, NY: Simon and Schuster.

[B26] Newell A. Simon H. A. (1972). Human Problem Solving. Englewood Cliffs, NJ: Prentice Hall.

[B27] Newen A. Bruin L. D. Gallagher S. editors (2018). The Oxford Handbook of 4E Cognition. Oxford: Oxford University Press. 10.1093/oxfordhb/9780198735410.001.0001

[B28] Plebe A. Kooij J. F. Rosati Papini G. P. Da Lio M. (2021). “Occupancy grid mapping with cognitive plausibility for autonomous driving applications,” in Proceedings of the IEEE/CVF International Conference on Computer Vision (ICCV) (Virtual Conference), 2934–2941. 10.1109/ICCVW54120.2021.00328

[B29] Poirier P. Chicoisne G. (2008). “A framework for thinking about distributed cognition,” in DrorHarnad:2008 (Amsterdam), 25–43. 10.1075/bct.16.03poi

[B30] Rumelhart D. E. McClelland J. L. (eds.). (1986). Parallel Distributed Processing: Explorations in the Microstructure of Cognition. Cambridge, MA: MIT Press. 10.7551/mitpress/5236.001.0001 25087578

[B31] SAE (2021). Taxonomy and Definitions for Terms Related to Driving Automation Systems for On-Road Motor Vehicles. SAE.

[B32] Smart P. (2017). Extended cognition and the internet-a review of current issues and controversies. Philos. Technol. 30, 357–390. 10.1007/s13347-016-0250-2 32010552 PMC6961510

[B33] Vanholme B. Gruyer D. Glaser S. Mammar S. (2011). “A legal safety concept for highly automated driving on highways,” in 2011 IEEE Intelligent Vehicles Symposium (IV) (Baden-Baden), 563–570. 10.1109/IVS.2011.5940582

[B34] Wang J. Huang H. Li K. Li J. (2021). Towards the unified principles for level 5 autonomous vehicles. Engineering 7, 1313–1325. 10.1016/j.eng.2020.10.018

[B35] Xing Y. Lv C. Cao D. Hang P. (2021). Toward human-vehicle collaboration: Review and perspectives on human-centered collaborative automated driving. Transp. Res. Part C 128:103199. 10.1016/j.trc.2021.103199

